# Chorioamnionitis disrupts erythropoietin and melatonin homeostasis through the placental-fetal-brain axis during critical developmental periods

**DOI:** 10.3389/fphys.2023.1201699

**Published:** 2023-07-20

**Authors:** Yuma Kitase, Nethra K. Madurai, Sarah Hamimi, Ryan L. Hellinger, O. Angel Odukoya, Sindhu Ramachandra, Sankar Muthukumar, Vikram Vasan, Riley Sevensky, Shannon E. Kirk, Alexander Gall, Timothy Heck, Maide Ozen, Benjamin C. Orsburn, Shenandoah Robinson, Lauren L. Jantzie

**Affiliations:** ^1^ Division of Neonatal-Perinatal Medicine, Department of Pediatrics, Johns Hopkins University School of Medicine, Baltimore, MD, United States; ^2^ Division of Pediatric Neurosurgery, Department of Neurosurgery, Johns Hopkins University School of Medicine, Baltimore, MD, United States; ^3^ Department of Pharmacology and Molecular Sciences, Johns Hopkins University School of Medicine, Baltimore, MD, United States; ^4^ Department of Neurology, Johns Hopkins University School of Medicine, Baltimore, MD, United States; ^5^ Kennedy Krieger Institute, Baltimore, MD, United States

**Keywords:** inflammation, perinatal brain injury, neurorepair, placenta, neural-immune, neuroimmunomodulation

## Abstract

**Introduction:** Novel therapeutics are emerging to mitigate damage from perinatal brain injury (PBI). Few newborns with PBI suffer from a singular etiology. Most experience cumulative insults from prenatal inflammation, genetic and epigenetic vulnerability, toxins (opioids, other drug exposures, environmental exposure), hypoxia-ischemia, and postnatal stressors such as sepsis and seizures. Accordingly, tailoring of emerging therapeutic regimens with endogenous repair or neuro-immunomodulatory agents for individuals requires a more precise understanding of ligand, receptor-, and non-receptor-mediated regulation of essential developmental hormones. Given the recent clinical focus on neurorepair for PBI, we hypothesized that there would be injury-induced changes in erythropoietin (EPO), erythropoietin receptor (EPOR), melatonin receptor (MLTR), NAD-dependent deacetylase sirtuin-1 (SIRT1) signaling, and hypoxia inducible factors (HIF1α, HIF2α). Specifically, we predicted that EPO, EPOR, MLTR1, SIRT1, HIF1α and HIF2α alterations after chorioamnionitis (CHORIO) would reflect relative changes observed in human preterm infants. Similarly, we expected unique developmental regulation after injury that would reveal potential clues to mechanisms and timing of inflammatory and oxidative injury after CHORIO that could inform future therapeutic development to treat PBI.

**Methods:** To induce CHORIO, a laparotomy was performed on embryonic day 18 (E18) in rats with transient uterine artery occlusion plus intra-amniotic injection of lipopolysaccharide (LPS). Placentae and fetal brains were collected at 24 h. Brains were also collected on postnatal day 2 (P2), P7, and P21. EPO, EPOR, MLTR1, SIRT1, HIF1α and HIF2α levels were quantified using a clinical electrochemiluminescent biomarker platform, qPCR, and/or RNAscope. MLT levels were quantified with liquid chromatography mass spectrometry.

**Results:** Examination of EPO, EPOR, and MLTR1 at 24 h showed that while placental levels of EPO and MLTR1 mRNA were decreased acutely after CHORIO, cerebral levels of EPO, EPOR and MLTR1 mRNA were increased compared to control. Notably, CHORIO brains at P2 were SIRT1 mRNA deficient with increased HIF1α and HIF2α despite normalized levels of EPO, EPOR and MLTR1, and in the presence of elevated serum EPO levels. Uniquely, brain levels of EPO, EPOR and MLTR1 shifted at P7 and P21, with prominent CHORIO-induced changes in mRNA expression. Reductions at P21 were concomitant with increased serum EPO levels in CHORIO rats compared to controls and variable MLT levels.

**Discussion:** These data reveal that commensurate with robust inflammation through the maternal placental-fetal axis, CHORIO impacts EPO, MLT, SIRT1, and HIF signal transduction defined by dynamic changes in EPO, EPOR, MLTR1, SIRT1, HIF1α and HIF2α mRNA, and EPO protein. Notably, ligand-receptor mismatch, tissue compartment differential regulation, and non-receptor-mediated signaling highlight the importance, complexity and nuance of neural and immune cell development and provide essential clues to mechanisms of injury in PBI. As the placenta, immune cells, and neural cells share many common, developmentally regulated signal transduction pathways, further studies are needed to clarify the perinatal dynamics of EPO and MLT signaling and to capitalize on therapies that target endogenous neurorepair mechanisms.

## 1 Introduction

Perinatal brain injury (PBI) is a leading cause of long-term morbidity and neurodevelopmental impairment in children (Kochanek et al., 2012; Novak et al., 2018). In recent years, modern medical advances in neonatal and perinatal critical care have improved the survival rate of preterm infants at earlier gestational ages (Larroque et al., 2008; Kyser et al., 2012). Despite this, concomitant advances in neuroscience have failed to effectively reduce the burden of disability associated with brain injury in this patient population. Indeed, more than half of those infants surviving PBI have chronic neurological conditions such as cerebral palsy, cognitive delay, epilepsy, sensory-motor disability, and deficits of attention (Volpe, 2009a; Nosarti et al., 2012; Anderson, 2014; Fant et al., 2014). Thus, the continued refinement of our understanding of the pathophysiological mechanisms of brain and neural-immune injury in infants is essential. Similarly, the identification of new strategies for neural repair and restoration, including therapies directed at improving developmental homeostasis and immunomodulation, is required. (Hodnett et al., 2010; Chen et al., 2016).

For a large proportion of infants with PBI, central nervous system (CNS) injury begins *in utero* with inflammation/infection (chorioamnionitis/CHORIO) and/or hypoxia-ischemia. CHORIO is the most common abnormality found in placentae from very preterm infants (Lee J. et al., 2013; Lee SM. et al., 2013; Chau et al., 2014). The placental dysfunction caused by CHORIO affects the entire maternal-placental-fetal axis (Redline, 2009). CHORIO affects placental permeability and blood flow, facilitating hypoxia-ischemia and transmission of inflammation to fetuses of all gestational ages. Indeed, the placental-fetal-brain axis plays an important role in neural development, and alterations or disruptions lead to chronic neurological deficits (Dammann and Leviton, 1997; Dammann and Leviton, 2014; Fant et al., 2014; Williams et al., 2017). In preterm infants, PBI can be diffuse and is known as encephalopathy of prematurity (Volpe, 2009b). PBI leads to impaired structural and functional connectivity in developing neural networks, and changes in brain network topology resulting in multiple motor, cognitive, and neuropsychiatric disabilities (Counsell et al., 2008) and lasting neural-immune injury (Lin et al., 2010; Huggard et al., 2020; Kitase et al., 2021).

Inflammation throughout the placental-fetal-brain axis and neural-immune aberrations after CHORIO are prominent factors in the pathophysiology of PBI. The associated signal transduction and specific molecules have been identified as therapeutic targets (Robinson et al., 2018a; Jantzie et al., 2018; Yellowhair et al., 2018; Yellowhair et al., 2019a; Yellowhair et al., 2019b; Kitase et al., 2021; Gall et al., 2022). For example, therapies such as erythropoietin (EPO) and melatonin (MLT) have been studied individually, and as a cocktail, in preterm infants (Yawno et al., 2017; Robinson et al., 2018a; Robinson et al., 2018b; Lee et al., 2019; Juul et al., 2020; Song et al., 2021; Wood et al., 2021; Jantzie et al., 2022). As monotherapy, both have been investigated in clinical trials to ameliorate CNS injury (Juul et al., 2008; Ohls et al., 2014; Fauchère et al., 2015; Ohls et al., 2016; Wu et al., 2016; Carloni et al., 2017). Like EPO, MLT has favorable pharmacokinetics and an acceptable safety profile (Buscemi et al., 2006; Gitto et al., 2013; Merchant et al., 2013; Andersen et al., 2016; Carloni et al., 2017). In clinical trials for preterm infants, MLT has been used for neuroprotection and neonatal sepsis (El-Kabbany et al., 2020; Garofoli et al., 2021).

EPO is an important molecule for neurorepair and immunomodulation as it has anti-apoptotic, antioxidant, and anti-inflammatory properties (Villa et al., 2003; Kumral et al., 2004; Maiese et al., 2008; Juul et al., 2009), alongside cytoprotective effects on endothelial cells, glial cells, and neurons (Mazur et al., 2010; Xiong et al., 2011; Gonzalez et al., 2013; Jantzie et al., 2015a; Ott et al., 2015). EPO affects neuronal differentiation (Park et al., 2006), neuroblast migration (Tsai et al., 2006), oligodendrogenesis (Iwai et al., 2010; Mazur et al., 2010; Jantzie et al., 2013), oligodendrocyte maturation (Sugawa et al., 2002; Mazur et al., 2010; Jantzie et al., 2013), myelination (Mazur et al., 2010; Jantzie et al., 2013; Jantzie et al., 2014a; Jantzie et al., 2016; Jantzie et al., 2018), and axon health (Hellewell et al., 2013; Jantzie et al., 2014b). EPO receptors (EPOR) play an important and critical role in development and tissue repair after injury (Mazur et al., 2010; Jantzie et al., 2019). Present in the brain early in gestation, EPO and EPOR are localized to neurons, oligodendrocytes, astrocytes, and choroid plexus. Both ligand and receptor expression persist after birth (Juul et al., 1999). After PBI, endogenous EPO levels transiently increase (Teramo and Widness, 2009; Logan et al., 2014; Sweetman et al., 2017; Teramo et al., 2018). Similarly, there can be a sustained upregulation of EPOR on many cells (Assaraf et al., 2007; Mazur et al., 2010; Messier and Ohls, 2014; Robinson et al., 2016). However, brain injury during development can persistently increase EPOR, without a sustained increase in EPO ligand production (Mazur et al., 2010). This mismatch can lead to cell death. MLT similarly has protective properties with antioxidant, anti-inflammatory, free radical scavenging and anti-apoptotic effects through receptor-mediated signaling, and non-receptor-mediated actions in brain, placenta, and immune cells. (Reiter et al., 2010; Tosini et al., 2014; Tarocco et al., 2019). Preterm infants, however, are at a developmental disadvantage in terms of its supply (Kennaway et al., 1992; Kennaway et al., 1996; Acuna-Castroviejo et al., 2014; Biran et al., 2019). MLT is synthesized at higher concentrations in the placenta than the pineal gland (Okatani et al., 1998; Man et al., 2017), and infants born before 34 weeks have lower blood levels of melatonin than those of mature infants, in part due to loss of placental sources. (Kennaway et al., 1992; Kennaway et al., 1996; Acuna-Castroviejo et al., 2014; Biran et al., 2019). Indeed, the rhythmic secretion of pineal MLT usually occurs around 2–3 months of age in full-term newborns (Commentz et al., 1996; Jan et al., 2007), but MLT secretion in preterm infants remains delayed and persists until 8–9 months of age (Biran et al., 2014; Biran et al., 2019; Ejaz et al., 2020)

In the present study, we evaluated EPO and MLT signaling throughout the placental-fetal-brain axis and modulation by CHORIO. We also evaluated SIRT1, HIF1α and HIF2α as upstream signaling partners and molecular convergence points between EPO and MLT pathways. (Chavez et al., 2006; Hou et al., 2011; Chen et al., 2012; Jantzie et al., 2022). We aimed to define changes in the expression of these important developmental regulators in the placenta-fetal-brain axis following injury. To accomplish this, we used an established model of CHORIO in rats (Jantzie et al., 2014a; Maxwell et al., 2015; Jantzie et al., 2018; Yellowhair et al., 2018; Yellowhair et al., 2019a; Yellowhair et al., 2019b; Kitase et al., 2021; Jantzie et al., 2022). This model yields cerebral palsy-like deficits in the mature CNS that mimic those of preterm survivors, with white matter injury, gliosis, spastic-like gait, poor social interaction, and cognitive impairment (Jantzie et al., 2014a; Maxwell et al., 2015; Jantzie et al., 2018; Yellowhair et al., 2018; Yellowhair et al., 2019a; Yellowhair et al., 2019b; Kitase et al., 2021; Jantzie et al., 2022). This model encompasses the fetal and systemic inflammatory response syndromes and placental pathology common in preterm infants with CHORIO, including acutely elevated IL-1β, TNF-α, IL-6, IL-10, and CXCL1 in serum and placental neutrophilia (Jantzie et al., 2014a; Maxwell et al., 2015; Yellowhair et al., 2018; Yellowhair et al., 2019a; Yellowhair et al., 2019b). The systemic inflammation associated with CHORIO causes PBMC hyper-reactivity at P7 (term-equivalent human age), P21 (toddler-equivalent human age), and throughout adulthood concomitant with profound immune dysregulation and changes in immune population dynamics throughout the lifespan (Yellowhair et al., 2018; Yellowhair et al., 2019a; Kitase et al., 2021). We hypothesized that there would be relative EPO and MLT expression changes after CHORIO that would mirror EPO and MLT fluctuations and alterations observed in human preterm infants including increases in EPO after injury, and relative melatonin and HIF2α deficiency. Similarly, we expected unique developmental regulation after injury that would reveal potential clues to the mechanisms, timing, and treatment of PBI.

## 2 Materials and methods

### 2.1 Animals

Sprague Dawley rat dams and litters were maintained in a temperature- and humidity-controlled facility with food and water available *ad libitum*. Animals were subject to a 12-h dark/light cycle, with lights on at 0800 h. All experiments were performed in strict accordance with protocols approved by the institutional Animal Care and Use Committee (ACUC) at the Johns Hopkins University School of Medicine. Protocols were developed and performed consistent with National Research Council and ARRIVE guidelines (Kilkenny et al., 2010).

### 2.2 *In utero* chorioamnionitis (CHORIO)

Pregnant Sprague Dawley rats underwent laparotomy on embryonic day 18 (E18) as previously reported (Jantzie et al., 2013; Jantzie et al., 2014a; Jantzie et al., 2014b; Jantzie et al., 2015a; Jantzie et al., 2015b; Maxwell et al., 2015; Jantzie et al., 2018). Under isoflurane anesthesia, uterine arteries were transiently occluded for 60 min to induce temporary placental insufficiency. Subsequently, an intra-amniotic injection of lipopolysaccharide (LPS 0111: B4, 4 μg/sac; Sigma-Aldrich, St. Louis, MO, United states) was administered to each amniotic sac, and the laparotomy was closed. The dam was allowed to recover and rat pups were born on embryonic day 22 (E22). Sham animals underwent a laparotomy (no uterine artery occlusion and no intra-amniotic LPS injection) with an equivalent duration of anesthesia. For each experiment described, the data represents true n (individual rats) from at least 4 different dams per condition. Balanced numbers of male and female offspring were used in every outcome measure.

### 2.3 Serum collection

Blood was collected on postnatal day (P) 2, P7, and P21 as a terminal procedure and centrifuged at 6,000 g for 15 min at 4°C. Serum was then removed and stored at −80°C for later analysis. Care was taken to avoid freeze-thaw cycles (Mitchell et al., 2005; Yellowhair et al., 2018).

### 2.4 Quantitative PCR

Placentae and forebrain were harvested at E19 (24 h following induction of CHORIO). Forebrain was also collected at P2, P7, and P21. Gene-of-interest primers and cDNA synthesized from 0.9 μg RNA were added to PowerUp™ SYBR™ Green Master Mix (Thermo Fisher Scientific, Waltham, MA), and run in triplicate on a Quant Studio™ 3 Real-Time PCR System (Thermo Fisher Scientific, Waltham, MA). To standardize transcripts from samples between experiments, cycle thresholds from samples of interest were compared to cycle threshold values from pooled E19 naïve samples, with gene-of-interest transcription normalized to ribosomal 18s endogenous control as previously published (Jantzie et al., 2013; Jantzie et al., 2014a; Jantzie et al., 2014b; Jantzie et al., 2015a). Primer sequences were verified with the Basic Local Alignment Search Tool (BLAST) for Nucleotides on the U.S. National Center for Biotechnology Information (NCBI) website. Primer sequences for EPO, EPO receptor (EPOR), Melatonin receptor 1 (MLTR1), Sirtuin 1 (SIRT1), HIF1α, HIF2α and 18S are listed in Table 1 (all from IDT Technologies, Coralville, Iowa). To ensure rigor, experimental replicates were run in triplicate and those cycle threshold (CT) values varying by greater than 0.25 standard deviations were excluded from all analyses.

**TABLE 1:** Primers used for qPCR.

Target	Primer sequence
EPO	(F)5′GCT CCA ATC TTT GTG GCA TC3′
	(R)5'ATC CAT GTC TTG CCC CCT A3′
EPOR	(F)5′GAC CCC AGC TCT AAG CTC CT3′
	(R)5′AGC CCC CTG AGC TGT AAT CT3′
SIRT1	(F)5′TGG CAC CGA TCC TCG AAC3′
	(R)5′CCG CTT TGG TGG TTC TGA AAG3′
Hif1α	(F)5′CCT ACT ATG TCG CTT TCT TGG3′
	(R)5′TGT ATG GGA GCA TTA ACT TCA C3′
Hif2α	(F)5′ATC AGC TTC CTG CGA ACA CA3′
	(R)5′CAG CCT CGG CTT CAG ATT CA3′
18S	(F)5′TCC CTA GTG ATC CCC GAG AAG T3′
	(R)5′CCC TTA ATG GCA GTG ATA GCG A3′

### 2.5 RNAscope

To localize single mRNA molecules of EPO, HIF1α, and HIF2α, RNAscope was performed on 12 μm paraformaldehyde-fixed frozen brain slices at the time points listed above. For each experiment, we used one positive (Rat *Polr2a*), and one negative (*E. coli DapB*) control probe, and probes against the mRNA of interest. *In Situ* hybridization (ISH) was performed according to manufacturer’s specifications and the RNAscope Multiplex Fluorescent Reagent Kit V2 protocol (ACD Biosciences, Newark, CA). Signal was detected with Opal 570. Confocal z stacks were acquired by blinded observers in the somatosensory cortex at the level of the dorsal hippocampus using a Nikon ECLIPSE Ti2 microscope (Tokyo, Japan) with a C2 system (variable emission confocal system coupled to a GaAsp detector). Gain parameters, zoom, pinhole size, step size, scan speed and resolution were uniform across scans.

### 2.6 Immunohistochemistry

Immunostaining for EPO was performed as previously described. (Mazur et al., 2010; Jantzie et al., 2013; Jantzie et al., 2014a; Jantzie et al., 2016; Jantzie et al., 2019). Specifically, rats were deeply anesthetized with ketamine and xylazine and perfused with 4% paraformaldehyde. Brains were then collected, and post-fixed in paraformaldehyde. After immersion in 30% sucrose solution, 50 μm, frozen, slide mounted, coronal sections were obtained and collected using a cryostat (Leica, Buffalo Grove, IL, United States). Slides were then washed and incubated with 0.5% Triton X, followed by blocking solution containing 10% normal goat serum in phosphate buffered solution (PBS). Primary antibodies against EPO protein (Epo clone B-4 sc-5290, Santa Cruz Biotechnology, 1:50, CA, United States), in blocking solution containing 0.5% Trition-X100 were incubated on sections overnight at 4°C. The next day, sections were rinsed, and incubated with species-appropriate biotinylated secondary antibodies for 1 h. This was followed by incubation in Fluorescein Avidin D (Vector Labolatories, CA, United States) for 1 h. Slides were then washed and coverslipped with ProlongTM Gold antifade reagent containing DAPI (Thermo Fisher Scientific, MA, United States).

### 2.7 Multiplex electrochemiluminescent immunoassay (MECI)

Serum EPO analyses were performed using an R-PLEX assay for the detection of rat EPO (R-PLEX Rat F231G, Meso Scale Diagnostics, Rockville, MD, United States). In accordance with the manufacturer’s specifications and as previously described (Maxwell et al., 2015; Robinson et al., 2016; Robinson et al., 2018b; Newville et al., 2020), serum was diluted to 1:2 and then loaded in duplicate with prepared standard onto a blocked and washed small-spot 96-well plate. EPO levels in brain and placentae were measured in the same manner after being adjusted to 300 µg tissue load (Yellowhair et al., 2019a; Robinson et al., 2018b; Robinson et al., 2016; Maxwell et al., 2015; Dammann et al., 2016; Kuban et al., 2015; Leviton et al., 2011; O'Shea et al., 2012). Plates were analyzed with a Quickplex SQ 120.

### 2.8 Liquid chromatography mass spectrometry (LCMS)

#### 2.8.1 Sample extraction

Proteins from serum were precipitated by the addition of 200 μL of LCMS grade acetonitrile to 50 μL of serum followed by vigorous vortexing for 30 s and centrifugation at 13,000 x g for 15 min at 4°C. The supernatant was pipetted off and dried with vacuum centrifugation (SpeedVac). The dried extract was resuspended in 20 μL of LCMS grade methanol followed by water bath sonication for 5 min at room temperature. The solution was again centrifuged at 13,000 x g for 15 min at 4°C and the supernatant was transferred to tapered autosampler vials for LCMS analysis.

#### 2.8.2 LCMS settings

Absolute quantification of melatonin was performed using a Dionex U3000 UHPLC system coupled to a Q Exactive “Classic” Mass Spectrometer using a 7-min gradient of 0.1% formic acid in LCMS grade water (Buffer A) and 0.1% formic acid in LCMS grade acetonitrile (Buffer B). All buffers were purchased from Fisher Scientific. A Poroshell 120 EC-18 2.1 × 50 mm column with 1.9 µm particles (Agilent) was used to separation with a consistent flowrate of 400 μL/min across the column maintained at 40°C. The gradient ramped from 5% B to 45% B in 3 min with a rapid ramp to 98% B in 0.5 min where it remained for 0.5 min before returning to baseline conditions for the remainder of the gradient. Eluting molecules were directly ionized into the Q Exactive at 3,500 V with a capillary temperature of 262°C and an accessory probe temperature of 425°C. Sheath gas and auxiliary gas pressures were set at 50 and 12.5 arbitrary units, respectively. A two-step method utilizing one full scan and one parallel reaction monitoring (PRM) scan were performed with the latter centered on the mass 233.1 with an isolation window of 2 Th. For the MS1 scan, all ions from 150 to 500 m/z were collected with a target of 3 × 10^6^ charges or a maximum injection time of 50 milliseconds at a resolution of 17,500 at m/z 200. For the PRM 2 × 10^5^ charges were acquired or a maximum of 50 milliseconds attempting to reach that charge number. All collected ions were fragmented at 20 eV.

#### 2.8.3 Data processing

The Thermo.RAW files were processed in the freely available Xcalibur QuanBrowser software (Thermo Fisher) with the area under the curve extracted for the PRM fragment ion of 233.1, 174.0915 using a 10 part per million (PPM) mass extraction window and a 15 s extraction window around the target elution time of 2.15 min. For concentration calculation, the area under the curve was compared to a serial dilution of melatonin obtained from the Johns Hopkins Hospital pharmacy diluted in methanol to an approximate concentration range of 1 picogram–60 picogram on column. A linear regression was automatically performed by the program and peaks were manually adjusted only in the case of obvious failures of the program to correctly integrate peak baselines.

### 2.9 Statistical analyses

Data are represented as mean ± standard error of the mean (SEM). Data were tested for normality with the Shapiro-Wilk Test. Parametric statistical differences between two groups were established with a *t*-test, and non-parametric differences between two groups were established with a Mann-Whitney *U*-test. GraphPad Prism 9.3.1 software was used for statistical analysis.

## 3 Results

### 3.1 Chorioamnionitis decreases placental EPO and MLTR1 mRNA expression

**FIGURE 1:**
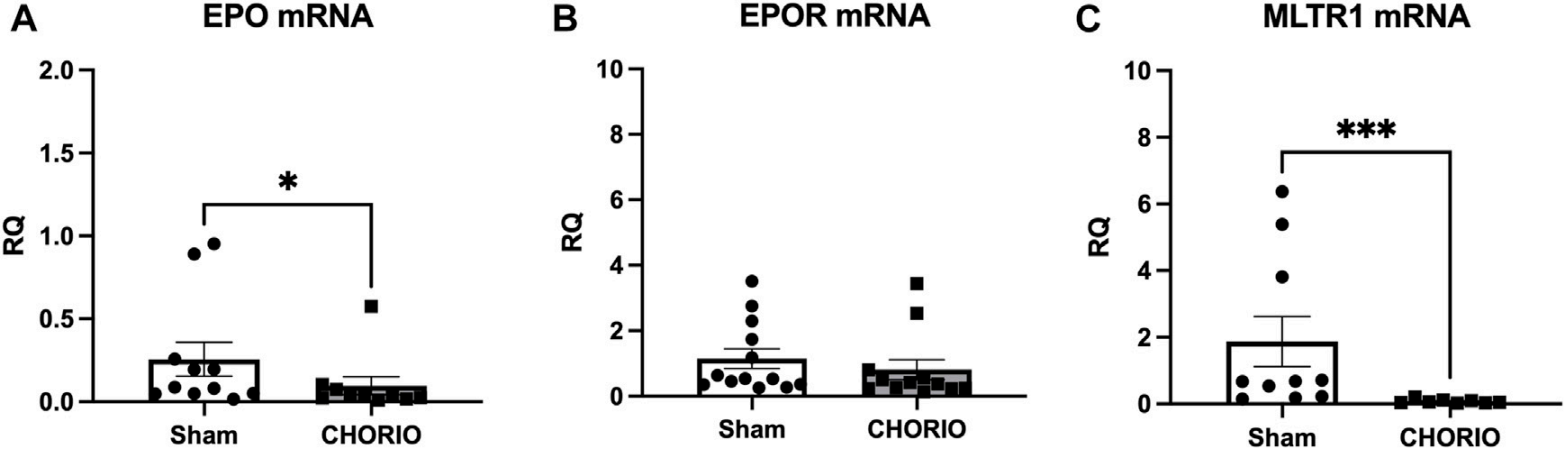
CHORIO decreases EPO and MLTR1 mRNA in placentae at E19. **(A)** In CHORIO placentae, EPO mRNA was significantly decreased compared to sham (Mann-Whitney *U*-test). **(B)** There was no significant difference between sham and CHORIO in EPOR mRNA (Mann-Whitney *U*-test). **(C)** MLTR1 mRNA levels were significantly lower in CHORIO placentae compared to sham (Mann-Whitney *U*-test). (*p < 0.05, ***p < 0.001).

The first stage of our investigation involved the assessment of EPO, EPOR, and MLTR1 mRNA, along with EPO protein expression in the placenta. In placentae at E19, 24 h following CHORIO, EPO mRNA was significantly decreased compared to sham (sham: 0.26 ± 0.10, *n* = 11, CHORIO: 0.10 ± 0.05, *n* = 10, *p* < 0.05, Mann-Whitney *U*-test) (Figure 1A). Interestingly, there was no companion alteration in EPO protein expression (sham: 1.19 ± 0.31 pg/mL, *n* = 6, CHORIO: 1.47 ± 0.34 pg/mL, *n* = 6, n.s., *t*-test) or EPOR mRNA expression (Figure 1B). Like EPO mRNA, assessment of MLTR1 mRNA expression in the placenta revealed a significant decrease following CHORIO compared to sham (sham: 1.87 ± 0.75, *n* = 10, CHORIO: 0.08 ± 0.02, *n* = 8, *p* < 0.001; Figure 1C, Mann-Whitney *U*-test). Acute reductions in these key signaling molecules is commensurate with profound inflammation from CHORIO (Jantzie et al., 2014a; Maxwell et al., 2015; Yellowhair et al., 2018; Yellowhair et al., 2019a; Yellowhair et al., 2019b; Kitase et al., 2021; Gall et al., 2022; Jantzie et al., 2022).

### 3.2 Chorioamnionitis acutely increases cerebral EPO, EPOR and MLTR1 mRNA

**FIGURE 2:**
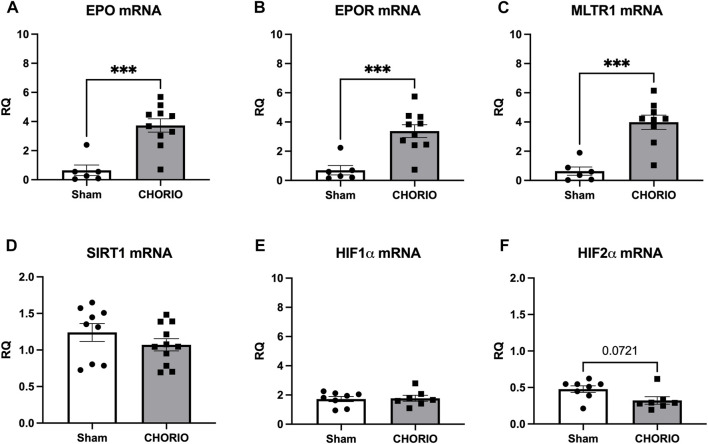
CHORIO markedly increases of EPO, EPOR, and MTLR1 mRNA in the fetal brain at E19. **(A, B)** EPO and EPOR mRNA levels were significantly elevated in CHORIO animals compared to sham (Mann-Whitney *U*-test). **(C)** MLTR1 mRNA levels in CHORIO brains was also significantly elevated compared to sham (*t*-test). **(D–F)** There was no significant differences in SIRT1, HIF1α, and HIF2α mRNA between sham and CHORIO. (***p < 0.001).

After assessing the placenta 24 h after the induction of CHORIO, we next examined the fetal brain at the same timepoint. Evaluation of E19 brains revealed significant increases in EPO and EPOR mRNA in CHORIO compared to sham (EPO sham: 0.65 ± 0.36, *n* = 6, CHORIO: 3.73 ± 0.46, *n* = 10, *p* < 0.001, Mann-Whitney *U*-test, Figure 2A; EPOR, sham: 0.70 ± 0.32, *n* = 6, CHORIO: 3.38 ± 0.44, *n* = 10, *p* < 0.001, Mann-Whitney *U*-test; Figure 2B). Notably, no cerebral increase in EPO protein was observed (sham: 0.43 ± 0.10 pg/mL, *n* = 9, CHORIO: 0.41 ± 0.19 pg/mL, *n* = 7, n.s., *t*-test). However, cerebral MLTR1 levels were also significantly increased in CHORIO brains compared to sham controls (sham: 0.64 ± 0.28, *n* = 6, CHORIO: 3.98 ± 0.49, *n* = 9, *p* < 0.001; Figure 2C, *t*-test). Unlike the increases in EPO, EPOR and MLTR1 mRNA, no significant change in SIRT1, HIF1α, HIF2α mRNA were observed in CHORIO brains compared to sham (Figures 2D–F). These data highlight the tissue-specific changes that occur in the acute period after CHORIO, with differential expression between the placenta and the fetal brain.

### 3.3 Chorioamnionitis differentially impacts cerebral levels of key developmental molecules in the postnatal period

**FIGURE 3:**
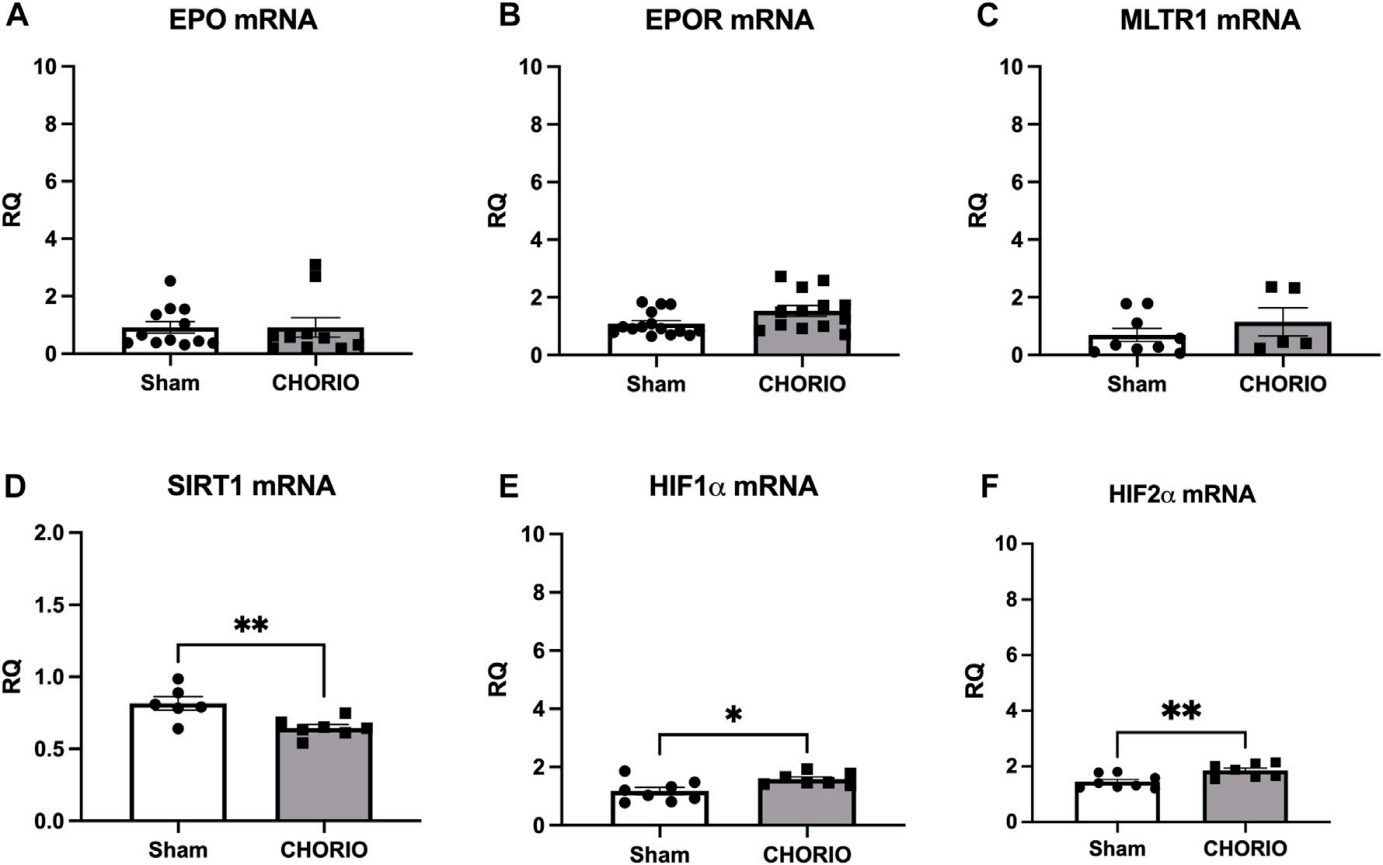
CHORIO Induces SIRT-1 Deficiency, and increases HIF1α and HIF2α at P2 but does not induce changes of EPO, EPOR, or MTLR1 mRNA. **(A–C)** CHORIO does not induce significant alterations in EPO, EPOR, nor MLTR1 mRNA in the P2 CHORIO brain compared to sham (Mann-Whitney *U*-test). **(D)** SIRT1 mRNA expression is reduced in CHORIO brains compared to sham controls (*t*-test). **(E, F)** HIF1α and HIF2α mRNA levels is elevated in CHORIO brains compared to sham (*t*-test) (*p < 0.05, **p < 0.01).

After assessing acute changes in EPO, EPOR, MLTR1, SIRT1, HIF1α and HIF2α in the fetal brain, we expanded our time course to the postnatal period from P2-P21 to assess cerebral injury-induced mRNA expression dynamics. At P2, a preterm equivalent time point approximately 5 days after CHORIO, EPO, EPOR, and MLTR1 mRNA expression were all stable and unchanged between CHORIO and sham groups. SIRT1 mRNA expression in the brain, however, was significantly decreased (sham: 0.82 ± 0.05, *n* = 6, CHORIO: 0.64 ± 0.02, *n* = 7, *p* < 0.01, Figure 3D, *t*-test) with increased HIF1α (sham: 1.18 ± 0.13, *n* = 8, CHORIO: 1.58 ± 0.08, *n* = 7, *p* < 0.05, Figure 3E, *t*-test) and HIF2α mRNA (sham: 1.45 ± 0.09, *n* = 8, CHORIO: 1.85 ± 0.09, *n* = 7, *p* < 0.05, Figure 3F, *t*-test) in CHORIO brains compared to sham.

**FIGURE 4:**
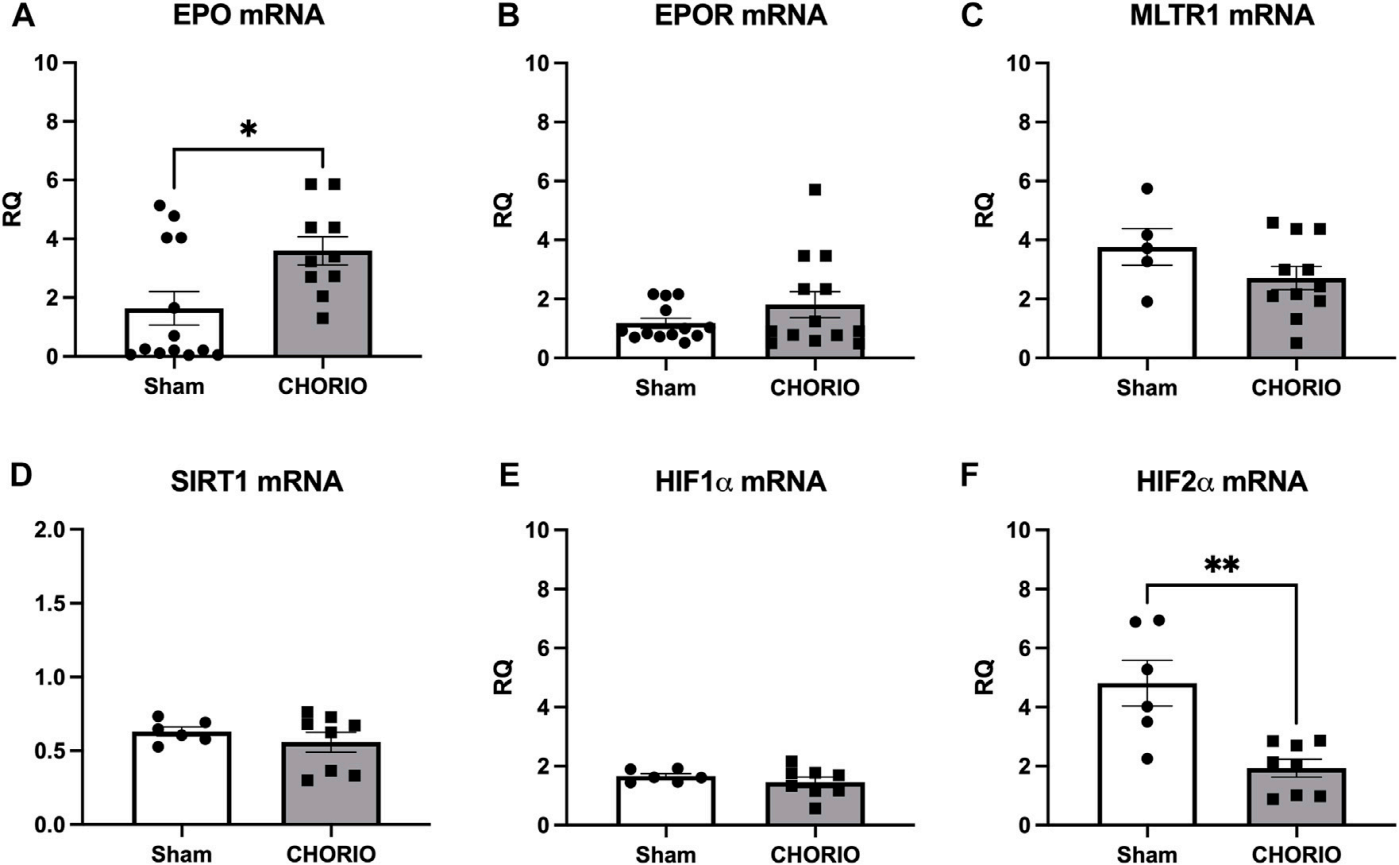
CHORIO differentially changes cerebral EPO and HIF2α levels at P7. **(A)** EPO mRNA was significantly increased in CHORIO brains compared to sham (Mann-Whitney *U*-test). **(B–E)** There was no significant difference between sham and CHORIO in EPOR, MLTR1, SIRT1 and HIF1α mRNA **(B)** Mann-Whitney *U*-test, **(C–E)** *t*-test). **(F)** CHORIO decreased HIF2α mRNA compared to sham (*t*-test) (*p < 0.05, **p < 0.01).

**FIGURE 5:**
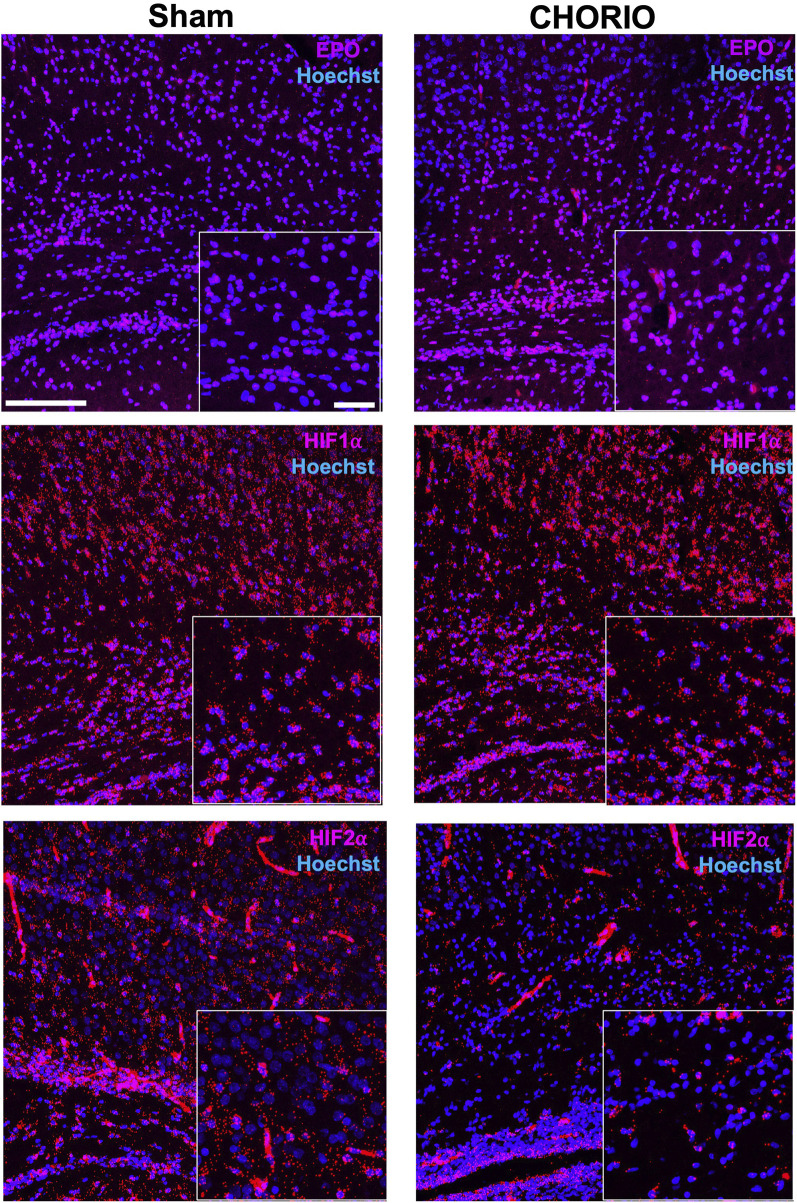
CHORIO increases EPO and decreases HIF2α mRNA spatial and temporal expression. Consistent with qPCR results at P7, a term-equivalent time point, RNAscope assays confirm changes in spatial and termporal localization of EPO mRNA with injury. Specifically, EPO mRNA is increased in the cortex of CHORIO brains compared to sham (Top Row). On the other hand, temporal and spatial localization of HIF1α mRNA does not differ between sham and CHORIO brains (Middle Row). However, HIF2α mRNA expression is deficient CHORIO brains compared to sham (Bottom Row). (Scale bars 100 μm and 10 μm inset).

**FIGURE 6:**
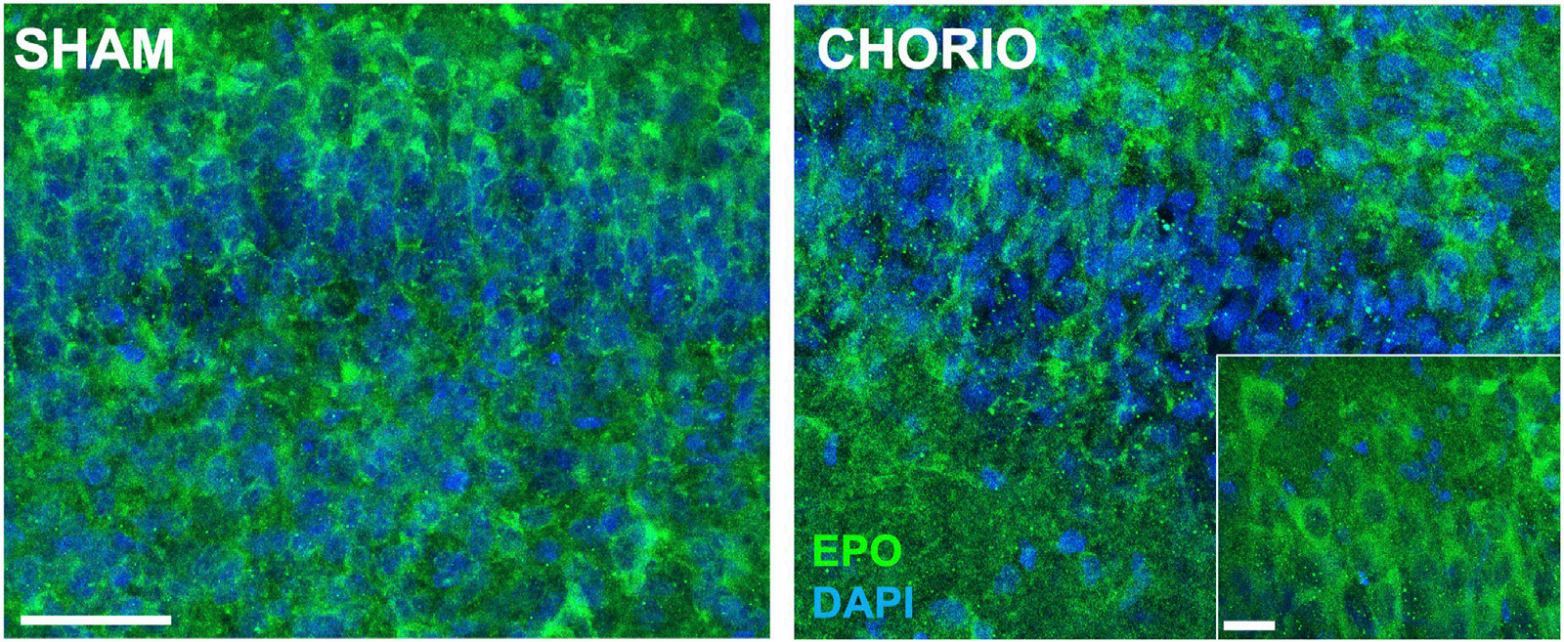
CHORIO increases EPO protein expression in the P7 brain. At P7, a term equivalent time point, EPO protein is increased in the hippocampus of CHORIO animals compared to sham consistent with expression level changes dependent on brain region, neural cell and injury response (Scale bars are 50 μm and 10 μm inset).

Extension of the time course to P7, a term equivalent time point, revealed increased EPO mRNA in the brains of CHORIO rats compared to sham (sham: 1.64 ± 0.57, *n* = 13, CHORIO: 3.59 ± 0.48, *n* = 10, *p* < 0.05, Figure 4A, Mann-Whitney *U*-test). This occurred without changes in EPOR, MLTR1, SIRT1, and HIF1α mRNA expression (Figures 4B–E). However, HIF2α mRNA expression was reduced in CHORIO brains compared to sham controls (sham: 4.82 ± 0.77, *n* = 6, CHORIO: 1.93 ± 0.30, *n* = 8, *p* < 0.01, Figure 4F, *t*-test). To add rigor, localize regional differences, and to examine dynamic cell-specific expression of the mRNAs, we performed RNAscope. Assessment of individual mRNAs revealed widespread increases in EPO with CHORIO, in the presence of decreased HIF2α throughout the somatosensory cortex (Figure 5). Consistent with previous publications, EPO protein was similarly expressed on neurons and oligodendrocytes with changes in expression level dependent on brain region and injury response (Figure 6).

**FIGURE 7:**
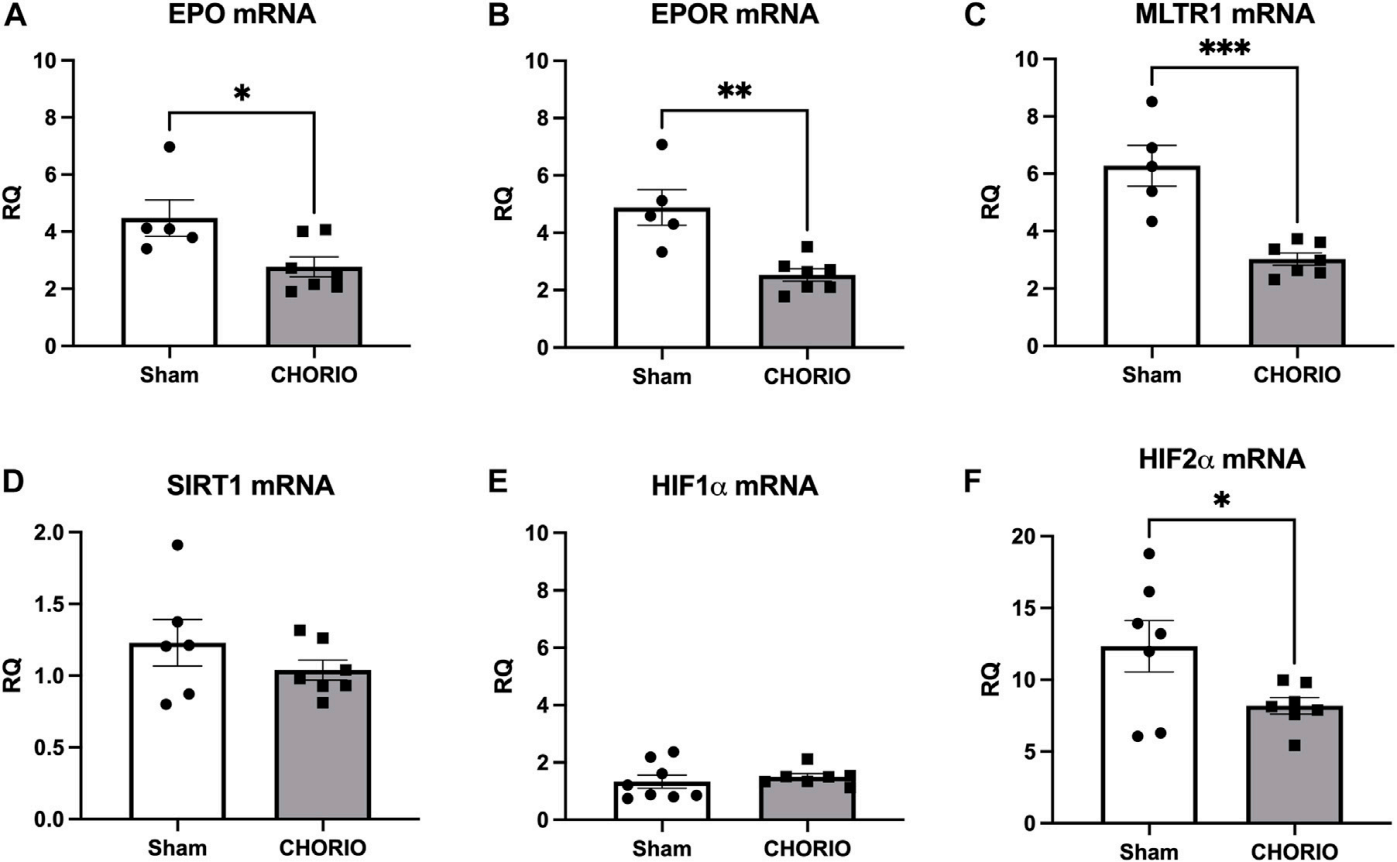
CHORIO causes profound decreases in cerebral EPO, EPOR, MLTR1, and HIF2α mRNA at P21. **(A, B)** EPO and EPOR mRNA levels were significantly decreased in CHORIO brains compared to sham **(A)** *t*-test, **(B)** Mann-Whitney *U*-test). **(C)** These changes were concomitant with decreased MLTR1 levels (*t*-test). **(D, E)** SIRT1 and mRNA showed no significant difference between sham and CHORIO (*t*-test). **(D, E)** SIRT1 and HIF1α mRNA showed no significant difference between sham and CHORIO (*t*-test). **(F)** HIF2α mRNA levels in CHORIO brains was significantly reduced compared to sham (*t*-test) (*p < 0.05, **p < 0.01 ***p < 0.001).

Notably at P21, a toddler equivalent time point, the pattern shifted considerably. Compared to sham, EPO mRNA (sham: 4.48 ± 0.64, *n* = 5, CHORIO: 2.77 ± 0.34, *n* = 7, *p* < 0.05, Mann-Whitney *U*-test, Figure 7A) and EPOR mRNA (sham: 4.88 ± 0.62, *n* = 5, CHORIO: 2.53 ± 0.22, *n* = 7, *p* < 0.01, *t*-test, Figure 7B) were significantly decreased in CHORIO brains. These changes were concomitant with decreased cerebral MLTR1 mRNA expression in CHORIO pups compared to sham (sham: 6.28 ± 0.70, *n* = 5, CHORIO: 3.03 ± 0.21, *n* = 7, *p* < 0.001, Figure 7C, *t*-test) and persistently reduced HIF2α mRNA expression (sham:12.34 ± 1.79, *n* = 7, CHORIO: 8.19 ± 0.58, *n* = 7, *p* < 0.05, Figure 7F, *t*-test). Thus, over the developmental time course from the third trimester through infancy and toddler stages of development, brain mRNA expression increases and then declines in the setting of CHORIO, emphasizing the complex pattern of changes in the injured, developing brain.

### 3.4 Perinatal brain injury causes mismatch in brain and serum EPO levels concomitant with variable melatonin levels

**FIGURE 8:**
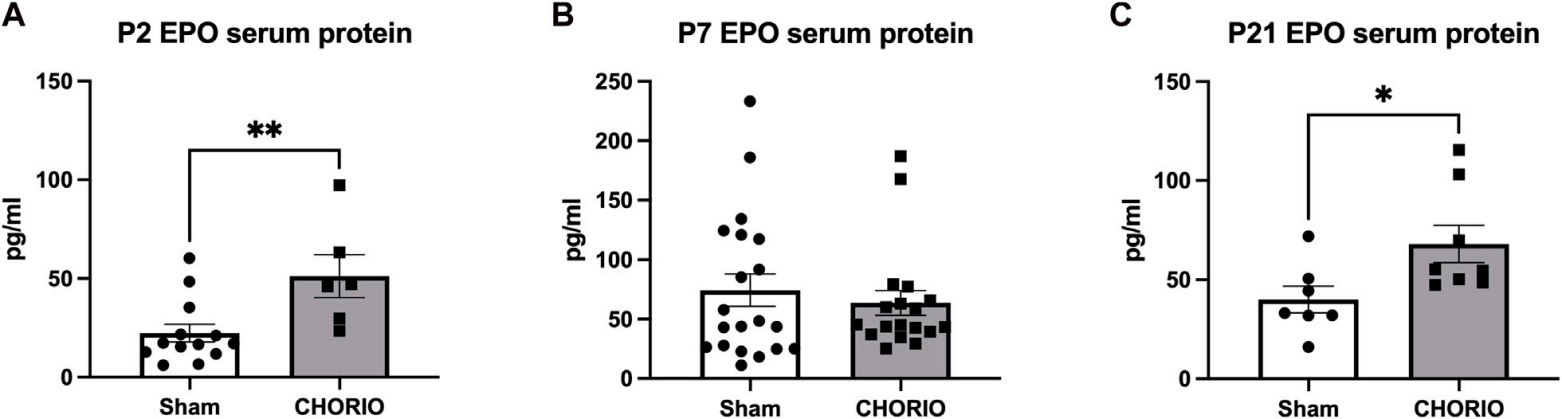
CHORIO Changes Serum EPO Levels. **(A)** Serum levels of EPO protein was significantly higher in CHORIO animals at P2 compared to sham (Mann-Whitney *U*-test). **(B)** By P7, there were no differences between sham and CHORIO groups apparent (Mann-Whitney *U*-test). **(C)** At P21, the EPO serum protein in CHORIO was again significantly higher than sham (Mann-Whitney *U*-test). (*p < 0.05, **p < 0.01).

**FIGURE 9:**
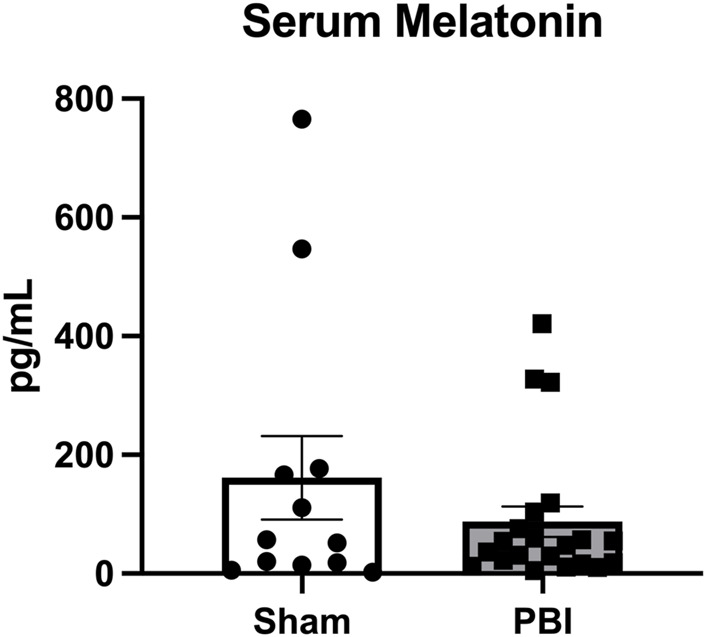
Melatonin levels in serum are variable between animals and treatment groups. As measured by liquid chromatography mass spectrometry, MLT levels were variable among individual animals independent of injury status.

Given the compartment-specific, and tissue-specific regulation of these key hormones, we next evaluated EPO and MLT protein expression in the serum. Unlike what was observed in placenta and brain tissue, serum EPO protein levels were markedly elevated across the developmental time course. Specifically, serum EPO protein was significantly higher in CHORIO animals at P2 compared to sham (sham: 22.3 ± 4.5 pg/mL, *n* = 13, CHORIO: 51.1 ± 10.9 pg/mL, *n* = 6, *p* < 0.01, Figure 8A). At P7, serum EPO levels in controls were elevated compared to P2 (P2: 22.3 ± 4.49 pg/mL, *n* = 13, P7: 74.33 ± 13.7 pg/mL, *n* = 20, *p* < 0.01, Mann-Whitney *U*-test), despite no observed significant difference between sham and CHORIO (Figure 8B). Interestingly, serum EPO levels were significantly higher in CHORIO pups at P21 compared to sham (sham: 40.0 ± 6.7 pg/mL, *n* = 7, CHORIO: 68.1 ± 9.4 pg/mL, n = 8, *p* < 0.05, Mann-Whitney *U*-test, Figure 8C) mirroring the CHORIO-induced increase at P2 and uniquely opposite to brain EPO mRNA expression at the same time point. Consistent with the clinical literature (Kennaway et al., 1992; Kennaway et al., 1996; Biran et al., 2019; Perrone et al., 2023), MLT levels were variable among individual animals independent of injury (161.5 ± 70.2 pg/mL, *n* = 12 vs. 87.4 ± 25.8 pg/mL, *n* = 21, *p* = ns, Mann-Whitney *U*-test, Figure 9). In summary, serum EPO and MLT levels do not necessarily reflect the complex pattern of mRNA expression noted in end organ brain tissue.

## 4 Discussion

Melatonin and erythropoietin are unique, developmentally regulated endogenous hormones essential to many body systems. Here, we studied changes throughout the placental-fetal-brain axis along an extended developmental time course. Our results delineate the complex, developmental, ligand-receptor-mediated, and tissue-specific regulation of these neuroimmunomodulatory molecules. Using an established preclinical model of PBI secondary to CHORIO with robust inflammation through the placenta-fetal brain axis (Maxwell et al., 2015; Robinson et al., 2018a; Jantzie et al., 2018; Yellowhair et al., 2019a; Kitase et al., 2021; Gall et al., 2022), we found that CHORIO also confers a sustained impact on EPO, EPOR, MLTR1, and HIF2α mRNA and protein from late gestation through early childhood. Notably, EPO and MLT signaling is multifaceted, and context dependent. We have previously shown that EPO/EPOR signaling is dependent on cell type and defined by developmental timing and injury response (Mazur et al., 2010; Jantzie et al., 2013; Jantzie et al., 2014a; Jantzie et al., 2015a; Jantzie et al., 2016). During neurodevelopment, EPO has multiple critical roles for most lineages including neurons (Tsai et al., 2006; Fernandez Garcia-Agudo et al., 2021), oligodendrocytes (Iwai et al., 2010; Jantzie et al., 2013), astrocytes (Tang et al., 2013), microglia and pericytes (Mazur et al., 2010; Fernandez Garcia-Agudo et al., 2021). Importantly, EPO’s protective properties require adequate SIRT1 expression (Hou et al., 2011; Wang et al., 2013; Li et al., 2019). Specifically, conditional mutants with SIRT1 knock-down fail to respond to exogenous EPO as wild-type animals do after injury. (Wang et al., 2013; Robinson and Jantzie, 2022).

MLT also acts on SIRT1 to increase its levels and shifts hypoxia-inducible factor (HIF) from HIF1α to HIF2α (Kudová et al., 2016). SIRT1 deficiency contributes to dysregulated HIF2α (hypoxia-inducible factor-2 alpha) (Chen et al., 2012), and HIF2α drives astrocytic production of EPO during neurodevelopment and immune maturation (Chavez et al., 2006; Semenza, 2019). During an acute hypoxic challenge, HIF1α and HIF2α functions overlap (Semenza, 2019; Packer, 2020; Packer, 2021). In contrast, in the setting of chronic inflammation, as reported in children with cerebral palsy (Lin et al., 2010; Zareen et al., 2020a; Zareen et al., 2020b), HIF1α and HIF2α functions diverge markedly. The shift to HIF2α contributes to immune cell health because HIF1α promotes toxicity, fibrosis, and inflammatory responses, while HIF2α provides nutritional and anti-inflammatory support (Packer, 2020; Robinson and Jantzie, 2022). Our data support that CHORIO induces a SIRT-1 deficiency at a preterm equivalent age concomitant with distinct, acuity-dependent EPO ligand- EPO receptor mRNA mismatch after CHORIO with accompanying changes in HIF2α. Specifically, we report deficient HIF2α mRNA expression at term equivalent (P7) and toddler equivalent (P21) developmental timepoints supporting longer term alterations in the setting of ongoing inflammation. We also report differential circulating levels of EPO and MLT in the serum compared to brain emphasizing the importance of both tissue levels and serum expression when evaluating injury and therapeutic response (Juul et al., 2020; Wood et al., 2021). Interestingly, MLT-SIRT1-HIF2α deficiency may contribute to the attenuated effect of exogenous EPO seen in preterm infants, and it is possible that increasing SIRT1 levels could allow for exogenous MLT to synergize with exogenous EPO to promote recovery after early brain damage (Robinson et al., 2018a; Jantzie et al., 2018; Robinson and Jantzie, 2022).

The data presented herein emphasizes the importance of EPO ligand-receptor mismatch and the increased demand for MLT following CHORIO. In addition to the changes in EPO, EPOR, SIRT1, HIF1α, and HIF2α observed, we also found that CHORIO induced placental reductions in EPO and MLTR1 mRNA expression. This was distinct from the time-dependent changes in cerebral MLTR1 mRNA expression. Maternal MLT plays a key role in fetal growth and in the early stages of neurodevelopment (Conway et al., 1997; Yu et al., 2017). Typical receptors for MLT regulation of circadian rhythm include MLTR1 and MLTR2 (Dubocovich, 2007; Zlotos et al., 2014), which are classical G-protein-linked receptors that inhibit adenylate cyclase (Zlotos et al., 2014). Placental trophoblasts synthesize melatonin and express its receptors (Lanoix et al., 2008). In placenta, MLTR1 receptors are involved in the regulation of apoptosis (Soliman et al., 2015). In the fetal brain, MLTR1 has been found in the leptomeninges, cerebellum, thalamus, hypothalamus, and brainstem (Thomas et al., 2002). Moreover, MLTR1 is found in the hippocampus, frontal cortex, and other brain regions that are important for cognition and memory (Ekmekcioglu, 2006). Although melatonin plays a key role in development, preterm infants are at a developmental disadvantage in terms of its supply (Muñoz-Hoyos et al., 2007; Merchant et al., 2013; Biran et al., 2019). MLT is synthesized in higher concentrations within the placenta and numerous other locations compared to the pineal gland (Okatani et al., 1998; Man et al., 2017; McCarthy et al., 2019). The rhythmic secretion of endogenous melatonin usually appears around 2–3 months of age after full-term birth (Commentz et al., 1996; Jan et al., 2007). However, infants born before 34 weeks have lower blood levels of melatonin (Biran et al., 2019), and melatonin secretion in preterm infants is delayed (Biran et al., 2014). We found CHORIO increases MLTR1 mRNA expression at E19, with subsequent decreases at P21, corresponding to human toddler age. This lag in recovery of MLTR1 levels suggests that MLT, when used for neurorepair in the developing CNS, is likely needed for an extended period to cover periods of developmental fluctuation, the multiple phases of PBI pathophysiology and innate recovery. Indeed, therapeutic strategies that maintain brain EPO, EPOR, and MLT homeostasis together may be needed as part of an extended regimen to achieve lasting brain protection. Notably, white matter injury in preterm infants progresses over an extended time course with concomitant gliosis and sustained elevations in key systemic inflammatory proteins throughout the lifespan with tertiary pathophysiology and permanently altered neural-immune developmental trajectory (Lin et al., 2010; Chau et al., 2014; Yellowhair et al., 2019a; Kitase et al., 2021).

This study is not without its limitations. Melatonin levels are difficult to measure, and LCMS -based evaluation of serum melatonin was used here. Future studies should include expanded time courses and direct brain melatonin measurements. MLT signaling also occurs independent of membrane bound MLTR1, and likely involves nuclear receptors. Since both EPO and MLT signaling occur in the placenta, immune cells, and brain, further studies are needed to elucidate the perinatal immune dynamics of EPO, MLT, SIRT1, and HIF2α signaling and to exploit the potential of endogenous therapeutic agents. The present study was conducted in a genetically homogeneous rodent background after a single stimulus and highlights the need to tailor diagnostic and therapeutic approaches to human infants on an individual basis with attention to initiating insults. In addition, since mRNA may not directly reflect actual protein expression or activity, degradation, phosphorylation, acetylation or other post-translational modification in the context of brain and placental injury, more detailed interpretation of homeostasis after CHORIO would be possible with additional studies of protein dynamics and function.

In conclusion, EPO and MLT drive signaling pathways that enhance sustained neuroimmunomodulation and promote microenvironmental homeostasis in the developing brain. CHORIO induces lasting effects on EPO, MLTR1, SIRT1, and HIF2α mRNA and protein expression suggestive of differential effects on EPO and MLT neurorepair capacity dependent on injury and developmental stage. Maintenance of MLT, SIRT1, and HIF2α homeostasis could modulate endogenous EPO levels and may drive signal transduction that maximizes neurorepair compared to EPO dosing in restricted, acute, and isolated regimens. Given the ligand-receptor mismatch found in preterm infants, and the supplementation of endogenous MLT signaling, the combination of EPO and MLT could be a reasonable and indispensable therapeutic strategy for infants with brain injury, as could therapies that support HIF2α.

## Data Availability

The raw data supporting the conclusion of this article will be made available by the authors, without undue reservation.
